# A mystery solved: human high-frequency middle-ear motion

**DOI:** 10.21203/rs.3.rs-5248220/v1

**Published:** 2024-10-29

**Authors:** Hideko Nakajima, Christopher McHugh, Michael Ravicz, Paul Secchia, Lukas Graf, Nam-Hyun Cho

**Affiliations:** Harvard Medical School, Massachusetts Eye and Ear; Massachusetts Eye and Ear; Massachusetts Eye and Ear; Harvard Medical School; Massachusetts Eye and Ear; Massachusetts Eye and Ear; Harvard Medical School, Massachusetts Eye and Ear

**Keywords:** Stapes, Sound Transmission, Optical Coherence Tomography (OCT), middle ear, human hearing

## Abstract

The middle ear transforms sound from low-impedance external air to high-impedance cochlear fluid. However, the human stapes – the input to the cochlea – has been reported to have minimal or no motion above ~ 4 kHz. For decades, this lack of observed high-frequency stapes motion has been puzzling, as it is inconsistent with our ability to hear up to 20 kHz. In this study we address this mysterious discrepancy. Here, we succeed in measuring robust high-frequency stapes motion up to 20 kHz in fresh human cadaveric ears. Furthermore, when stapes motion is robust at high frequencies, these ears also show robust cochlear partition motion measured *in situ*. The ability to measure robust high-frequency stapes and cochlear motion represents an advancement in the field. To preserve high-frequency sound transmission, we modify surgical technique to prevent loosening of the ossicular chain. This implies that similar modifications in otologic surgery for patients may better preserve high-frequency hearing. The importance of this is that the ability to understand speech in noisy environments and sound localization requires high-frequency hearing.

## Introduction

Humans can hear from 20 Hz to about 20 kHz, yet previous reports of middle-ear motion have consistently showed that the transmission of sound through the middle ear seems to not reach such high frequencies^1, 2, 3, 4, 5, 6, 7, 8, 9, 10, 11^. Measurements of stapes motion, the cochlear input in human cadaveric specimens, suggest that the middle ear is not capable of transmitting appreciable sounds above 4 kHz, but these sounds are certainly heard by human subjects. This apparent discrepancy between sound transmission and sound perception has been a mystery in the field of hearing research for decades.

Knowledge about middle-ear function in human has relied on experiments in fresh human cadaveric specimens. The use of such specimens has also revolutionized our understanding of middle-ear pathology, reconstruction, and design of prostheses^3, 12, 13^. Because middle-ear motion in fresh cadaveric specimens is similar to the live ear up to about 3 kHz, stapes motion, the input to the cochlea, is often used as a proxy for hearing^13, 14, 15, 16^. However, the relationship between stapes velocity measurements and hearing has not been established for frequencies above about 4 kHz – frequencies important for speech perception and sound localization^17^.

**Figure 1:**
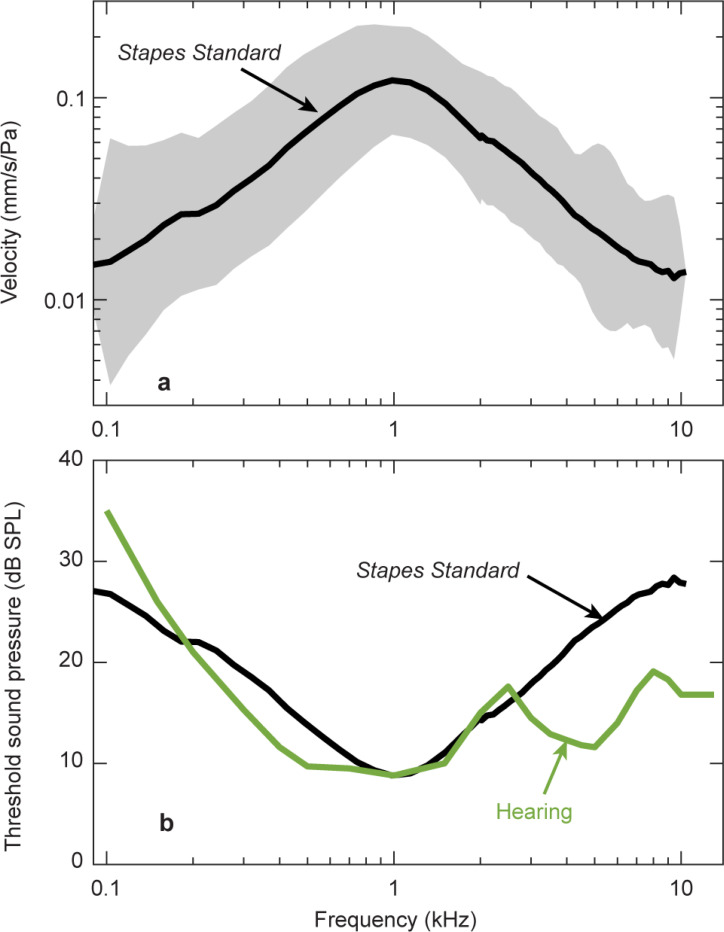
**(a)** *Stapes Standard* velocity was measured with 1D LDV referenced to ear-canal sound pressure. Mean (black line) and 95% confidence interval (shading) from13 publications (Rosowski, 2007) became an ASTM international *Stapes Standard*. **(b)** Comparison of thresholds between Stapes Standard in black and human hearing in green. Threshold of *Stapes Standard* is the sound pressure level needed to produce a stapes velocity of 200 nm/s, calculated from data in (a). Threshold for stapes velocity is chosen to match the threshold of hearing at 1 kHz. At frequencies above 3 kHz, *Stapes Standard* threshold is higher than reported hearing threshold (Killion, 1982).

The link between stapes velocity and hearing in human is generally accepted, especially below 3 kHz where robust stapes velocity measurements have been reported. A standard for stapes velocity was established based on several published studies (Fig. 1a), becoming the American Society for Testing and Material (ASTM) international consensus standard^7^. This standard shows a decrease in stapes velocity above 1 kHz (Fig. 1a) of about 20 dB per decade. When inverted to show sound threshold for a specific stapes velocity, this threshold of stapes velocity can be compared with the threshold of hearing. Sound thresholds for stapes velocity versus frequency has a similar shape as the human threshold of hearing below ~ 2.5 kHz (Fig. 1b).

At higher frequencies this similarity of threshold shape between stapes velocity and hearing does not hold; human hearing is better above ~ 2.5 kHz than would be predicted by stapes velocity. The hearing threshold (green curve) in Fig. 1b is lower than the stapes velocity threshold (black curve). Somehow human hearing is more sensitive at high frequencies than what measurements of stapes motion seem to indicate.

**Figure 2:**
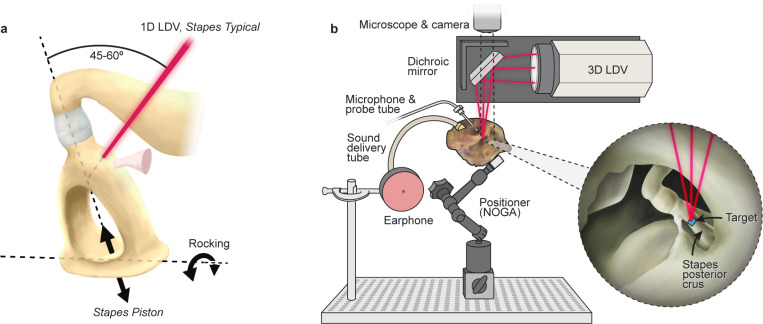
**(a)** Illustration of the human stapes showing relevant motions and measurement direction. The *Stapes Piston* motion is shown by black arrows near the footplate. *Stapes Typical* motion is measured with a 1D LDV (laser beam shown with red) that targets the stapes posterior crus from a direction about 45–60^0^ relative to the piston direction. Rocking motion about the long axis of the footplate is shown by the curved arrows.**(b)** Experimental setup for 3D LDV to estimate *Stapes Piston velocity*. The specimen was glued to a positioner. The 3D LDV was mounted on translation stages for easy focusing. Sound was delivered from the earphone to the ear canal and a probe tube microphone measured ear-canal sound pressure near ear drum. The 3D LDV laser beams were reflected by a dichroic mirror to focus vertically downward (–*z* direction) onto a target on the stapes posterior crus. *Stapes Piston velocity* was estimated with coordinate transformation.

Although past studies have attempted to explain this discrepancy between human stapes motion and hearing ability at high frequencies, there has been no satisfactory explanation. It is a difficult problem with several parts:

1. Do stapes velocity measurements represent cochlear input? Piston motion of the stapes at the oval window is considered the effective input to the cochlea. However, one-dimensional (1D) stapes velocity is usually measured with laser Doppler velocimeter (**LDV**) at one point on the stapes posterior crus or footplate with 45–60 degree angle relative to the piston direction (Fig. 2a). Here we refer to the 1D measurement as *Stapes Typical*.
2. Stapes motion is known to be complex, with varying modes of motion above a few kHz. Can non-piston stapes motion at high frequencies, such as rocking motion (Fig. 2a), contribute to cochlear input? Studies have tried to answer this question, but results were inconclusive^4, 16, 18, 19^.
3. Is it possible that standard clinical surgical methods to access the middle ear (also used to prepare fresh cadaveric temporal bone for experimentation) result in loosening the ossicular chain? A study that intentionally loosened the ossicular joints showed a reduction in high-frequency middle-ear sound transmission^20^.

To address the questions above, we measure high-frequency stapes motion in the piston direction. Additionally, we determine sound transmission into the cochlea at high frequencies by measuring the motion of the cochlear partition. To do this, we used novel techniques in six fresh human cadaveric specimens:

1. We determine *Stapes Piston* velocity for a wide range of frequencies. As shown in Fig. 2b, we use a 3D LDV to measure stapes velocity along three axes simultaneously, then use coordinate transformation to compute *Stapes Piston* velocity.
2. We determine sound transmission to the cochlea. While measuring *Stapes Typical* with 1D LDV (Fig. 2a), we simultaneously measure *Cochlear Partition* motion *in situ* in an intact cochlea with optical coherence tomography (**OCT**) vibrometry. This determines the correspondence between motion of the stapes and cochlear partition and whether high-frequency sound is transmitted to the cochlea.
3. We modify surgical preparation of our specimens to minimize contact with or displacement of the ossicular chain to prevent loosening of the ossicular chain.

Measurements of stapes motion above 4 kHz as an input reference to cochlear response measurements have both great scientific and clinical utility. Robust high-frequency stapes velocity measurements also pave a path to test new implantable prostheses targeted to improving frequencies important in speech perception, sound transmission during surgery, and many other valuable applications^21, 22^. Moreover, we report human cochlear partition motion *in situ* through an intact round-window membrane with OCT for a wide frequency range.

## Results

### Stapes velocity can be robust at high frequencies

**Figure 3:**
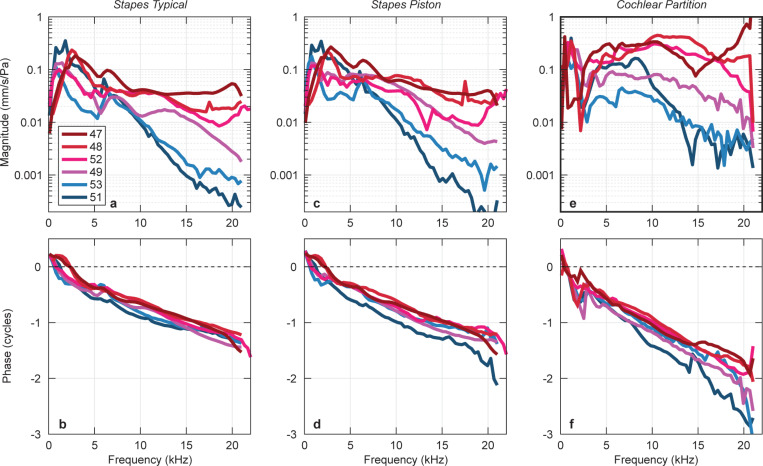
Magnitude and phase frequency response of stapes and cochlear partition velocities normalized by ear-canal sound pressure from 6 ears. Ears with large high-frequency velocitymagnitudes are plotted with shades of red, while ears with small high-frequency magnitudes are in shades of blue. **(a & b)** *Stapes Typical* measured with 1D LDV. **(c & d)** *Stapes Piston*computed from 3D LDV measurements. **(e & f)** *Cochlear Partition*velocity measured with OCT at the junction of the bridge and BM (location shown in Fig. 4). Legend identifies the experiment and temporal bone number.

We measured *Stapes Typical* velocity with a 1D LDV from a direction about 45–60^0^ with respect to the piston motion (Fig. 2a). To minimize measuring the stapes rocking component, the laser direction was approximately in-line with the plane defined by the two stapes crura. Figure 3a plots the *Stapes Typical* magnitude with respect to frequency in 6 ears. (A linear x-axis frequency scale details the high frequencies.) At low frequencies, magnitudes peak between 0.8 and 3 kHz, similar to the published standard (Fig. 1a)^7^. Above 7–10 kHz, *Stapes Typical* magnitude in two ears (shades of blue) decreased rapidly with frequency, similar to the prior standard measurements^7, 23, 24^. However, in four ears (shades of red/pink), *Stapes Typical* magnitudes remained above ~ 0.01 mm/s/Pa above 7–10 kHz. *Stapes Typical* phases plotted in Fig. 3b were similar across ears and to previous studies, with a group delay of approximately 60 μs, corresponding to the delay between ear-canal sound pressure near the eardrum and the motion of the stapes^24^.

The Stapes Piston motion (illustrated in Fig. 2a) was computed from 3D LDV measurements (Fig. 2b)^25^. Overall, Stapes Piston velocity magnitudes (Fig. 3c) had similar frequency responses as the Stapes Typical (Fig. 3a). The phases of *Stapes Piston* (Fig. 3d) were similar to *Stapes Typical*, with the exception of ear 51 phase accumulating an additional half cycle at high frequencies. *Stapes Piston* group delays (~ 70 μs) were similar to *Stapes Typical* group delays. In general, for each ear the frequency responses of Stapes Typical and the *Stapes Piston* velocities were similar.

### In Situ OCT measurement of cochlear partition motion in the intact cochlea:

**Figure 4:**
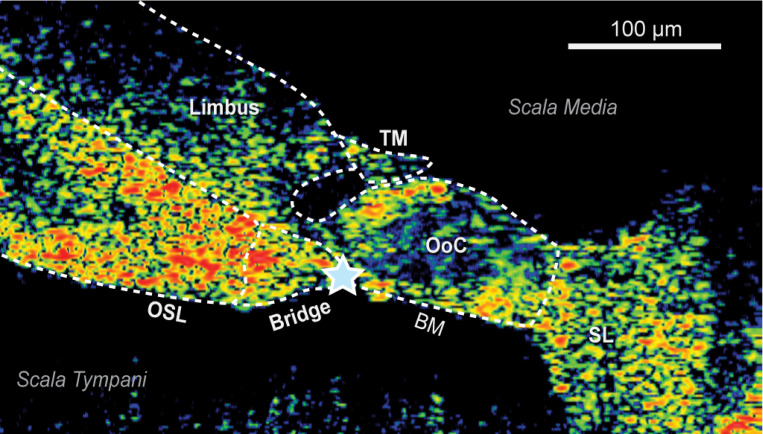
*In situ* OCT image of the cross section of the human cochlear partition viewed through the intact round-window membrane (round window would be below this image). The color scale indicates the reflectivity of structures: red and orange show high reflectivity, blue shows low reflectivity. From left to right (medial to lateral), cochlear structures can be distinguished and outlined: the osseous spiral lamina (OSL) at the left in orange/yellow extends to the right where it connects to the soft-tissue bridge. The bridge then connects to the basilar membrane (BM) at the bridge-BM junction (indicated by blue star), where *Cochlear Partition* motion was evaluated. Other labeled structures are the limbus, tectorial membrane (TM), organ of Corti (OoC), and spiral ligament (SL).

To determine sound transmission to the cochlea, we used OCT to image and measure motion of the cochlear partition in response to sound in the same ears with stapes velocity measurements described above. We imaged cochlear structures through the intact round-window membrane while keeping the cochlea intact and undisturbed. Figure 4 shows a cross-sectional “B-scan” image of a cochlear partition as viewed with OCT. Black areas represent fluid of scala media above and scala tympani below the colored areas of the cochlear partition. Bright orange colors of the cochlear partition represent optically high reflection, while the darker blue represent low reflection. Several cochlear microstructures can be distinguished, including: the osseous spiral lamina (**OSL**), the basilar membrane (**BM**), the bridge between the OSL and BM, as well as the limbus, tectorial membrane, and organ of Corti. In this study we evaluated motion at the junction between the bridge and BM, at the location indicated by the star. Previously, we found with Laser Doppler Vibrometry (LDV) that this Bridge-BM junction region generally has the largest sound-induced displacement ^26^.

### At high frequencies, cochlear partition motion is large if stapes motion is large

*Cochlear Partition* transverse velocity normalized by ear-canal sound pressure near the Bridge-BM junction is shown in Fig. 3e&f. Overall, the magnitude of *Cochlear Partition velocity* across ears is similar to *Stapes Piston* and *Stapes Typical*. Above 3–10 kHz, ears with high *Cochlear Partition* magnitudes (red lines in Fig. 3e) were the same ears with high magnitude *Stapes Typical* and *Stapes Piston* (red lines in Fig. 3a&c). The ears with low *Cochlear Partition* magnitudes above 3–10 kHz (blue lines) were also the same ears with low *Stapes Typical* and *Stapes Piston* magnitudes. Compared to phases of *Stapes Piston* and *Stapes Typical, Cochlear Partition* phase (Fig. 3f) lagged more with group delay of ~ 95 μs between 5 and 15 kHz (slightly more than the delay of 83 μs reported previously with intracochlear pressure measurements at the vestibule^27^.

## Discussion

For decades, it has been a mystery that measurements of human stapes motion in cadavers have small magnitudes at high frequencies despite humans having good high-frequency hearing (Fig. 1b). Here we show that *Stapes Typical* (Fig. 2a) measured with 1D LDV in the manner of previous studies can indeed be larger at high frequencies than previously reported (Fig. 3a). To achieve high-frequency stapes motion, we use a modified surgical technique to access the ossicles.

Furthermore, *Stapes Piston* velocity calculated from our 3D LDV measurements, is similar to the *Stapes Typical*. Thus, the *Stapes Typical* measurement is a reasonable estimate of *Stapes Piston* even at high frequencies. Ears with robust high-frequency stapes motion are also associated with robust high-frequency sound transmission to the cochlea, as measured with *Cochlear Partition* motion with OCT vibrometry (Fig. 3e).

**Figure 5:**
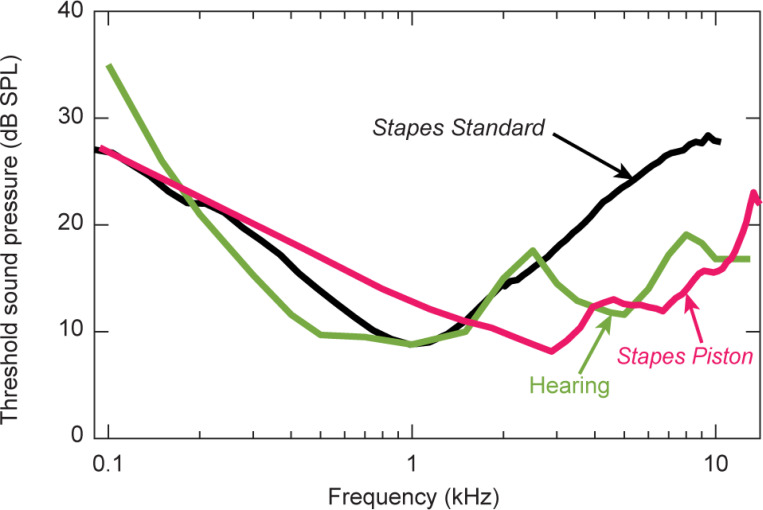
Relationship between stapes motion and hearing. Similar to Fig. 1b, *Stapes Standard*and *Hearing* thresholds are plotted with black and green. Additionally, the *Stapes Piston* threshold (mean from Fig. 3c red/pink curves with robust high-frequency motion) are plotted with pink. The threshold for *Stapes Piston* from the present study and *Hearing* threshold are similar, especially at frequencies above 2500 Hz. Stapes thresholds were the stimulus sound level required for 200 nm/s stapes velocity

The new robust high-frequency stapes measurements are consistent with robust human high-frequency hearing. In Fig. 5, the high-frequency threshold of hearing (green) is similar to threshold for *Stapes Piston* (pink), unlike the old threshold for *Stapes Standard* (black).

### Surgical modifications to preserve high-frequency stapes motion

In our previous work as well as in most studies, *Stapes Typical* (Fig. 2a, 1D LDV stapes motion measured 45–60^0^ angle from the piston motion) resembled the *Stapes Standard* frequency response shape (similar to Fig. 1a). Interestingly, though rare, there have been high-frequency stapes velocity measurements showing larger high-frequency motion than the standard of what is usually reported. Such data have been considered outliers, but may represent examples where high-frequency stapes motion were better preserved^23^.

We hypothesize that the method of standard surgical preparation of temporal bones often loosens the ossicular chain, thus compromising high-frequency stapes motion. In this study we perform a new surgical technique where we barely touch or disturb the ossicles and keep ossicular surrounding soft tissues intact. This surgical technique allows us to record high-frequency stapes motion in addition to high-frequency motion of the cochlear partition.

With this new surgical method, four of the six specimens had large *Stapes Piston* and *Stapes Typical* velocity magnitudes and smooth phases at high frequencies (see Fig. 3a-d). In Fig. 5, we plot the sound pressure necessary for a threshold of stapes velocity of 200 nm/s (as in Fig. 1b). Our new *Stapes Piston* threshold curve (pink curve in Fig. 5) has low thresholds at high frequencies, similar to the threshold of hearing curve (green). Modifying surgical technique can preserve high-frequency stapes motion and this motion is consistent with sound transmission to the cochlea and human hearing at high frequencies.

The preservation of high-frequency motion has important implications for studying cochlear mechanics. Previous studies that measured intracochlear sound pressures in fresh human ear specimens had magnitudes that greatly decreased with increase in frequency above 1–2 kHz^11, 23, 27, 28, 29^. These intracochlear pressure measurements had similar frequency responses of stapes motion and had low high-frequency motion. In a recent study, we began using our modified surgical technique, enabling large intracochlear scala vestibuli pressure at high frequencies^26^. In the present study, ears with large stapes motion (*Stapes Piston* and *Stapes Typical* velocities) at high frequencies also have large high-frequency *Cochlear Partition* motion (Fig. 3 red curves). If *Stapes Piston* and *Stapes Typical* have low motion at high frequencies, high-frequency *Cochlear Partition* are also low (Fig. 3 blue curves).

### Clinical implications

By avoiding manipulation of the ossicles during surgical preparation of our specimens, we can preserve high-frequency stapes motion. This implies that standard manipulation of the ossicles as part of otologic surgery may pose a risk of compromising high-frequency hearing. Therefore, understanding the effect of standard surgical practice on high-frequency sound transmission and possibly modifying surgical technique should be explored.

Although we tried to perform all our surgeries similarly, 2/6 ears had low stapes and cochlear motion at high frequencies. It is possible that we loosened the middle-ear ossicular chain during our surgical preparation. It is also possible that these ears originally had loose ossicular chains, which would be a source for high-frequency conductive hearing loss^20, 30^. Clinically, however, hearing loss above 4 kHz is assumed to represent sensorineural hearing loss. In some cases, this may be a misdiagnosis. High-frequency conductive loss can be missed because bone-conduction audiograms are not usually measured above 4 kHz. Developing high-frequency bone-conduction audiograms would help to differentiate between sensorineural and conductive high-frequency hearing. Wideband acoustic immittance measurements that measures the mechanics of the ear non-invasively has also been shown to determine loosening of the ossicles^20, 31, 32, 33, 34, 35^. However wideband acoustic immittance measurement is not generally used to diagnose ossicular pathology, but there is promising active research to translate this type of measurement in the clinic^36^. If loosening of the ossicular chain can be diagnosed, surgical repair, and therefore hearing restoration, could be possible.

## Methods

### Specimen preparation

Six cadaveric temporal bones were collected from donors with no known history of otologic disease. All specimens were obtained with permission (Mass General Brigham protocol # 2020P000508) from Massachusetts General Hospital. Specimens were obtained within 12–38 hours postmortem and were never exposed to chemical fixatives. Two of the specimens (SR52, 53) were used fresh soon after procurement, while the others were frozen immediately after procurement then thawed for experiments. All specimens were male, aged 44–82 years, and were right ears except for one. All specimens appeared free of disease and pathology during preparation. Care was taken to keep specimens moist and cool.

To prepare specimens, holes were made in the ear-canal wall to accommodate tubes for delivering and measuring ear-canal sound. A mastoidectomy allowed for access to the middle-ear cavity through an extended facial recess opening with facial nerve resected to visualize the stapes posterior crus for LDV and to obtain adequate exposure to the round window membrane for OCT vibrometry.

Great care was taken not to disturb the ossicular chain to prevent loosening the ossicular chain. The middle ear and inner ear were kept intact. Touching the ossicles with instruments was kept to a minimum, and suctioning tip was kept away from the ossicles. We placed a reflective target on the stapes posterior crus for LDV measurements while minimizing touching and moving the stapes. The stapedial tendon was preserved and almost all soft tissue including mucosal folds interfacing the ossicular chain was left undisturbed.

### Sound stimulation and response measurements

Tones between 0.1 and 21 kHz from an earphone speaker were delivered to the ear canal. The lateral end of the ear canal was open. Ear-canal sound pressure was measured with a probe-tube microphone with tip located 1–2 mm of the center of the tympanic membrane. Custom software running on a National Instruments PXI synthesized sound stimuli, measured ear-canal microphone responses and stapes LDV responses simultaneously, and synchronized the OCT vibrometry system (described below).

### Measurement of stapes velocity

Two methods were used to measure stapes velocity.

1. *Stapes Typical*: A single 1D LDV beam was focused on a reflective target on the stapes posterior crus using a direction as close to the stapes piston motion as possible (see Fig. 2a) and in the plane defined by the stapes crura to minimize measuring the contribution of stapes rocking motion. The orientation of the LDV laser beam relative to the specimen was noted and captured in photos to aid in positioning the specimen on the 3D LDV as described below. 1D LDV measurements were made concurrently with OCT vibrometry to determine *Cochlear Partition* motion (described below).
2. *Stapes Piston* and coordinate transformation: The 3D LDV has an optical head with three laser beams in a conical pattern 120 degrees apart. Laser beams were directed vertically downward (–*z* direction) as shown in Fig. 2b. The optical head was mounted on a motorized stage, and the entire setup was on an air table^37^. Camera acquired images in the horizontal *x–y* plane aided in determining measurement directions.

We oriented the specimen so the three laser beams were focused on the target on the posterior crus from the same direction as in the 1D LDV measurements (*Stapes Typical* defined as the *z-axis* direction). The angle between the 3D-LDV horizontal *y* axis and the *Stapes Piston* direction was measured to facilitate coordinate transformation to compute the *Stapes Piston* motion.

### Imaging and vibrometry of the cochlear partition with OCT

Cross-sectional images of the cochlear partition (Fig. 4) was obtained with the OCT system (GAN620C1, Thorlabs, Germany; 900-nm center wavelength, 46 kHz A-line scan camera frame rate, 2.2–8 μm resolution with 36 mm 0.055NA, 2x objective lens). Transverse displacement (direction nearly perpendicular to the bridge-BM) was measured at a specified point at the intersection of the basilar membrane and bridge (Fig. 4). Image acquisition was controlled and synchronized with the stimulus by our custom software.

### Course of experiment

On an air table inside a sound chamber, the specimen was kept moist and cool throughout the experiment with cooled saline to minimize deterioration of cochlear structures. The specimen was oriented to visualize the cross-sectional cochlear partition through the intact round window membrane to reveal structures of interest with OCT (Fig. 4). During cochlear partition displacement measurements with OCT, *Stapes Typical* was measured simultaneously with 1D-LDV (Fig. 2a). The 1D-LDV measurement direction relative to the stapes was recorded. Velocities of the cochlear partition and *Stapes Typical* were also measured with no stimulus present to estimate the noise floor. The specimen was then positioned for the 3D LDV so its orientation relative to the vertical 3D-LDV laser beam patterns was the same as for the 1D-LDV (z-axis) as described above to obtain *Stapes Piston*.

## Summary and conclusions

*Stapes Piston* velocity is robust to high frequencies of 10–20 kHz. The single-point LDV *Stapes Typical* velocity, measured similarly to previous publications on the stapes posterior crus with a 45–60^0^ angle from the piston motion, is similar to the *Stapes Piston* velocity. If performed in the manner described here, the *Stapes Typical* provides a reasonable representation of *Stapes Piston* to nearly 20 kHz.

Human *Cochlear Partition* motion measured by OCT vibrometry shows that sound transmission to the cochlea can be robust at high frequencies if *Stapes Piston* and *Stapes Typical* have robust high frequency motion. Conversely, specimens that exhibited poor Stapes Piston and *Stapes Typical* motion have poor *Cochlear Partition* motion at high frequencies.

With a change in surgical method where the ossicles are undisturbed and soft-tissue connections to ossicles are preserved, robust stapes motion can be obtained up to nearly 20 kHz. Such robust high-frequency stapes motion is consistent with the ability for humans to hear up to 20 kHz. This modification of surgical method to preserve transmission of high-frequency sound to the cochlea could influence change in the technique of clinical middle-ear surgery.
